# Form from Function, Order from Chaos in Male Germline Chromatin

**DOI:** 10.3390/genes11020210

**Published:** 2020-02-18

**Authors:** Peter J. I. Ellis, Darren K. Griffin

**Affiliations:** School of Biosciences and Centre for Interdisciplinary Studies of Reproduction, University of Kent, Giles Lane, Canterbury CT2 7NJ, UK; P.J.I.Ellis@kent.ac.uk

**Keywords:** spermatogenesis, chromatin, nuclear organisation, DNA oxidation, DNA fragmentation, epigenetic inheritance, histone retention, assisted reproduction, in vitro fertilisation

## Abstract

Spermatogenesis requires radical restructuring of germline chromatin at multiple stages, involving co-ordinated waves of DNA methylation and demethylation, histone modification, replacement and removal occurring before, during and after meiosis. This Special Issue has drawn together papers addressing many aspects of chromatin organization and dynamics in the male germ line, in humans and in model organisms. Two major themes emerge from these studies: the first is the functional significance of nuclear organisation in the developing germline; the second is the interplay between sperm chromatin structure and susceptibility to DNA damage and mutation. The consequences of these aspects for fertility, both in humans and other animals, is a major health and social welfare issue and this is reflected in these nine exciting manuscripts.

One of the most fundamental requirements in spermatogenesis is the need to develop male germ cells to undergo radical restructuring of their chromatin. Occurring at multiple stages before, during and after meiosis, it involves coordinated waves of DNA methylation and demethylation. It also involves histone modification, replacement and removal. In this Special Issue, we draw together novel studies and contemporary reviews addressing various aspects of chromatin organization and dynamics in the male germ line, and consider both humans and model organisms. Two major themes emerge from these exciting studies: the first being the functional significance of nuclear organization in the developing germline and the second is the interplay between sperm chromatin structure and DNA damage. The consequence of these aspects for fertility, both in humans and other animals, is a major health and social welfare issue.

Fernanda López-Moncada and colleagues address the question of whether chromosomal reorganization alters gene expression during meiotic prophase [1]. In particular, they show that Robertsonian fusions involving chromosomes bearing nucleolar organizing regions (NOR) perturb their normal organization and nucleolar functionality. In post-meiotic spermatids, Jonathan Riel and colleagues show that Sly deficiency is not the only reason for infertility in mice with deletions on their Y chromosome. Rather, it appears that some other Yq-encoded gene is likely to be required to allow Sly to bind to chromatin and to exert its normal regulatory functions [2].

Four studies examine chromosome organisation in mature sperm. First, Dimitris Ioannou and Helen Tempest show that, while chromosomes in human sperm do indeed to form hairpin loops, as predicted from studies in other species, their centromeres are not organized in the classic “chromocenter” arrangement seen in model species such as mice [3]. Second, Heather Fice and Bernard Robaire confirm that relative sperm telomere length does indeed decrease during ageing in rodents, but, crucially, only in inbred strains [4]. Moreover, the demonstration that relative telomere length changes as sperm pass through the epididymis is a novel one. Third, Ben Skinner and colleagues address the question of whether chromosome territory organization is conserved between species, demonstrating that mouse chromosomes have retained the same sub-nuclear “address” for over two million years of evolutionary history [5]. Finally, Alexandre Champroux and colleagues turn to the possible deleterious effect of oxidative damage on sperm DNA organization. The surprising finding is that territory organization is largely robust in response to this challenge, with the overall organization of the chromosome territories being maintained even in the face of oxidative DNA damage. However, this organization is then disrupted in response to the treatment, illustrated by the reducing agents, signifying that oxidative damage may perturb chromosome decondensation following fertilization [6].

The theme of DNA damage is covered extensively in our two review articles. While DNA damage is usually regarded as a pathological, abnormal process, Tiphanie Cavé and colleagues review the role of endogenous, naturally-occurring DNA strand breaks created during chromatin remodeling [7]. This is an emerging field with profound implications for our understanding of the processes generating structural variations and polymorphisms within the genome, and the male versus female bias of specific mutational signatures. In a similar vein, but with a more clinical focus, Jordi Ribas-Maynou and Jordi Benet take a look at the differential reproductive effects on male fertility of single and double strand sperm DNA damage, respectively [8]. By their account, single-strand DNA breaks are present as scattered break points throughout the genome, whereas double-strand DNA breaks are mainly localized and attached to the sperm nuclear matrix. Single strand breaks are related to oxidative stress and impede pregnancy rates, whereas double strand breaks may be related to a lack of meiotic DNA repair—or to genome reconfiguration by topoisomerases, as highlighted by Cavé and colleagues—and lead to increased miscarriage rates, low embryo quality and implantation failure during ICSI.

Finally, we are particularly proud of the use of novel methods for studying the interplay between chromatin structure and the susceptibility to DNA damage and mutation. Indeed, this Special Issue boasts three new methodological approaches with Sheryl Homa and colleagues comparing two means of measuring oxidative stress (concluding that both used in tandem are better than one in isolation) [9] and both the Skinner and Champroux papers taking novel approaches to quantify the localization of chromosome territories in asymmetrical nuclei [5,6].

Collectively, these papers serve to highlight the importance of understanding male germline chromatin organisation in order to appreciate how specific regions of the genome may well be exposed to different stressors, remodeled, and activated before or after others immediately following fertilization. This, in turn, has downstream effects on both male germline mutagenesis and for early embryonic development; with profound subsequent implications for understanding natural fertility and improving assisted reproduction techniques.

Taken together, this unique collection of studies will, we hope, serve as a benchmark for a deeper understanding of the fundamental mechanisms perpetuating our germline.
